# Designing the Essential Informational Needs of a Smartphone Application for Self‐Management of Patients With Inflammatory Bowel Disease

**DOI:** 10.1002/hsr2.70186

**Published:** 2024-11-18

**Authors:** Farkhondeh Asadi, Azamossadat Hosseini, Amir Hossein Daeechini

**Affiliations:** ^1^ Department of Health Information Technology and Management School of Allied Medical Sciences, Shahid Beheshti University of Medical Sciences Tehran Iran

**Keywords:** inflammatory bowel disease, informational needs, mobile application, needs assessment, self‐management

## Abstract

**Background and Aims:**

Inflammatory bowel disease (IBD) is a chronic digestive disease that has a detrimental effect on the quality of life of IBD patients. This study aims to identify the informational needs and design the essential informational needs for a smartphone application for the self‐management of IBD.

**Methods:**

This study was conducted in two stages and the informational needs of the patients were extracted in a questionnaire designed in three separate sections and given to 120 patients with UC and 60 patients with CD.

**Results:**

After a literature review and analysis of patient responses, it was found that Knowledge of the disease, Medication, Educational information, Complications, Diet & Nutrition, and Lifestyle habits are among the most important domains of informational needs of inflammatory bowel disease patients.

**Conclusion:**

Patients with IBD have many informational needs, and in this study, identifying these needs, can help improve the quality of life of these patients and be of interest to healthcare providers, designers, and developers of applications.

**Abbreviations**
CD: Crohn's disease
IBD: Inflammatory bowel disease
UC: Ulcerative colitis

## 1 Introduction

Inflammatory bowel diseases (IBD) are chronic gastrointestinal disorders caused by the immune system, including Crohn's disease (CD) and Ulcerative colitis (UC) [1]. CD can cause inflammation anywhere in the lining of the digestive tract, while UC causes long‐term inflammation in specific parts of the digestive tract (mainly the large intestine). These two diseases lead to chronic digestive system inflammation and cause many clinical manifestations in patients [2].

The incidence and prevalence of IBD are on the rise globally. Currently, Europe reports 2.5 million cases, while the United States has over one million individuals affected by this condition, signaling a growing health concern worldwide [3]. IBD incurs a high economic burden so that healthcare costs for IBD patients are estimated to be more than 1.7 billion dollars annually [4]. Symptoms such as frequent diarrhea, urgency, bleeding, and incontinence necessitate constant access to the toilet, food restrictions, and changes in the lifestyle of patients with IBD, profoundly impacting the patients' lives [5, 6, 7].

Due to the chronic and aggravating nature of IBD, this disease can harm various aspects of a patient's well‐being, including mental status, patients' nutrition, employment, and family planning [8, 9, 10]. Patients suffering from this disease often require multiple interventions and continuous monitoring, which can force them to deviate from their routine life and lead to mental and social disorders in these patients [11], such that patients with IBD are twice as likely to experience generalized anxiety disorder [12].

Differences in clinical manifestation patterns, side effects of the disease, the expansion of IBD education for healthcare professionals, and medication adherence are among the challenges hindering the provision of optimal clinical care for patients with IBD [13].

Due to the chronic nature of this disease (periods of recovery and relapse), patients with IBD are the ideal target group for self‐management [14]. Self‐management is a novel aspect of managing chronic diseases, enabling patients to follow and regulate their treatment based on a predetermined process and have greater control over their condition [15, 16, 17].

Self‐management and patient cooperation reduce the cost of healthcare and the disease side effects [18]. Patient unawareness of different domains of the disease decreases the level of trust in doctors and increases the patients' fear and anxiety, which can negatively impact the patient's self‐management. Doctors and healthcare staff should address this issue [19]. Therefore, providing accurate information regarding disease progression enables patients to properly comprehend their condition, manage it more effectively, and make informed decisions throughout their treatment [20].

A great demand for optimized healthcare and treatment exists to provide health information through smartphones and the continuous supply of information on the web [14]. Hamilton's study shows that patients with IBD are interested in getting up‐to‐date information about IBD to effectively control and manage their disease, and developed smartphone apps help them achieve this objective [21].

The success of self‐management smartphone apps depends on proper application design, and the first step is to identify the informational needs of patients with IBD. Furthermore, identifying the informational needs of patients facilitates informed decision‐making in the course of the disease [22].

The informational needs vary depending on the stages of the disease, from general information about the condition (at diagnosis) to medications and treatments (at relapse) and daily management of the disease [23]. Patients with CD need more information than patients with UC regarding pregnancy, medications, and lifestyle, indicating different informational needs between patients with UC and CD [20].

In general, developing health applications for patients with IBD is in the early stages, and these applications are yet to meet all the informational needs of patients with IBD [24]. Therefore, the present study aims to identify the informational needs and design the essential informational needs for a smartphone application for the self‐management of IBD.

## 2 Methods

### 2.1 Study Design

This applied descriptive study was conducted in two stages in 2023 to identify the essential informational needs in the design of the self‐management application for patients with IBD.

### 2.2 Stage A: Data Collection

A comprehensive literature review was conducted using relevant articles, specialized guidelines for IBD self‐management, and World Gastroenterology Organization (WGO) guidelines. In addition, databases such as Scopus, Google Scholar databases, PubMed, and Web of Science using the keywords “informational needs,” “needs assessment,” “mobile application,” “inflammatory bowel disease,” “ulcerative colitis” and “crohn's disease” searched until December 2023.

Specific criteria were defined for the inclusion of articles, the inclusion criteria included English articles whose full text was available and had sufficient details about the informational needs of patients with inflammatory bowel disease.

Conference articles, letters to the editor, review articles, and articles that lacked sufficient details about the informational needs of patients with inflammatory bowel disease were considered as exclusion criteria. Based on the established inclusion and exclusion criteria, the studies were independently reviewed by A.H.D. and F.A. were screened and selected.

### 2.3 Stage B: Identification and Selection of Data

After reviewing the retrieved studies, the essential informational needs and required capabilities of the smartphone application for the self‐management of patients with IBD were extracted and categorized in an Excel file.

Then, based on the research objectives, the informational needs extracted from the texts and studies were divided into six categories: Knowledge of the disease, Medication, Educational information, Complications, Diet & Nutrition, and Lifestyle habits.

A researcher‐made questionnaire (based on a 5‐point Likert scale) in three sections, including demographic and clinical information (10 questions), essential information needs (25 questions), and required capabilities of the mobile application for patients with IBD (15 questions) for two groups of patients with UC and CD were designed.

The questionnaire was examined in a specialized panel with five gastroenterologists and five health information management specialists. At the suggestion of one of the specialists in the specialized panel, an unstructured question was included at the end of the questionnaire to receive opinions and suggestions. Specialists added or removed no questions from the questionnaire. Two questions from the essential informational needs (educational information domain) and one from the required capabilities section of the mobile application were modified. After applying corrections and specialists' comments, the final version of the questionnaire with 50 questions was approved.

The opinions of 10 experts, including five gastroenterologists and five health information management specialists, who were all faculty members and had at least 5 years of work and teaching experience in the research field, were obtained to check the content validity of the questionnaire.

Besides, SPSS software version 26 was used to calculate Cronbach's alpha coefficient to measure the reliability of the questionnaire. Cronbach's alpha coefficient was calculated at 87.7% for the patient's demographic and clinical information section, 89.2% for the essential informational needs of patients with IBD, and 85.7% for the required capabilities section of the application.

In one of the gastroenterology centers affiliated with Tehran, the objectives of the research were explained by a member of the research team in an interview with 200 patients aged 18 to 60 with inflammatory bowel disease, and they were assured that their answers would remain confidential.

Informed consent was obtained from the patients, but 20 patients were excluded from the study due to regretting the research and incomplete completion of the questionnaire.

Finally, the paper version of the questionnaire was distributed and completed in person by one of the researchers (F.A) among 180 patients with IBD, including 120 patients with UC and 60 patients with CD who had sufficient skills to work with mobile phones.

The participants were asked to rate each of the items in the questionnaire.

The scores of the items were as follows: 5 = very important, 4 = important, 3 = no idea, 2 = slightly important, and 1 = unimportant.

Data analysis was done using descriptive statistics and SPSS software version 26.

## 3 Results

### 3.1 Demographic and Clinical Details of Participants

The demographic information and clinical characteristics of the participants are given in Table 1. Approximately 57.5% of 120 participants with UC and 55% of 60 participants with CD were women; the rest were men. 80% of patients with CD and 67.5% with UC had been diagnosed for over 5 years. While none of the patients with CD were over 50 years old, 15 patients with UC were between 50 and 60. Sixty percent of UC cases and 30% of CD cases were employed. Notably, more than 83.34% of 120 patients with UC and 95% of 60 patients with CD did not have a family history of IBD.

**Table 1:** Demographic and clinical details of participants (*n* = 180).

Variable	Value	Crohn's disease	Valid presence	Ulcerative colitis	Valid presence	IBD	Valid presence
Gender	Male	27	45%	51	42.5%	78	43.3%
	Female	33	55%	69	57.5%	102	56.7%
Age (years)	18–30	12	20%	27	22.5%	39	21.7%
	31–40	27	45%	48	40%	75	41.7%
	41–50	21	35%	30	25%	51	28.3%
	51–60	0	0%	15	12.5%	15	8.3%
Marital status	Single	9	15%	33	27.5%	42	23.3%
	Married	51	85%	87	72.5%	138	76.7%
Education status	High school or less	6	10%	18	15%	24	13.3%
	Diploma	24	40%	30	25%	54	30%
	Higher diploma/university	9	15%	11	9.2%	20	11.1%
	BS	18	30%	42	35%	60	33.3%
	MSc	3	5%	19	15.8%	22	12.3%
Employment status	Working	18	30%	72	60%	90	50%
	Homemaker	18	30%	24	20%	42	23.3%
	Retired	9	15%	9	7.5%	18	10%
	Unemployment	15	25%	15	12.5%	30	16.7%
Family history of IBD	Yes	3	5%	20	16.66%	23	12.8%
	No	57	95%	100	83.34%	157	87.2%
Duration of treatment	< 1	3	5%	18	15%	21	11.7%
	2–5	9	15%	27	22.5%	36	20%
	> 5	48	80%	75	62.5%	123	68.3%
History of surgery	Yes	21	35%	24	20%	45	25%
	No	39	65%	96	80%	135	75%
Disease duration (years)	< 1	3	5%	9	7.5%	12	6.6%
	2–5	9	15%	30	25%	39	21.7%
	> 5	48	80%	81	67.5%	129	71.7%

### 3.2 The Domain of Informational Needs of Patients With Ibd

**Figure 1:**
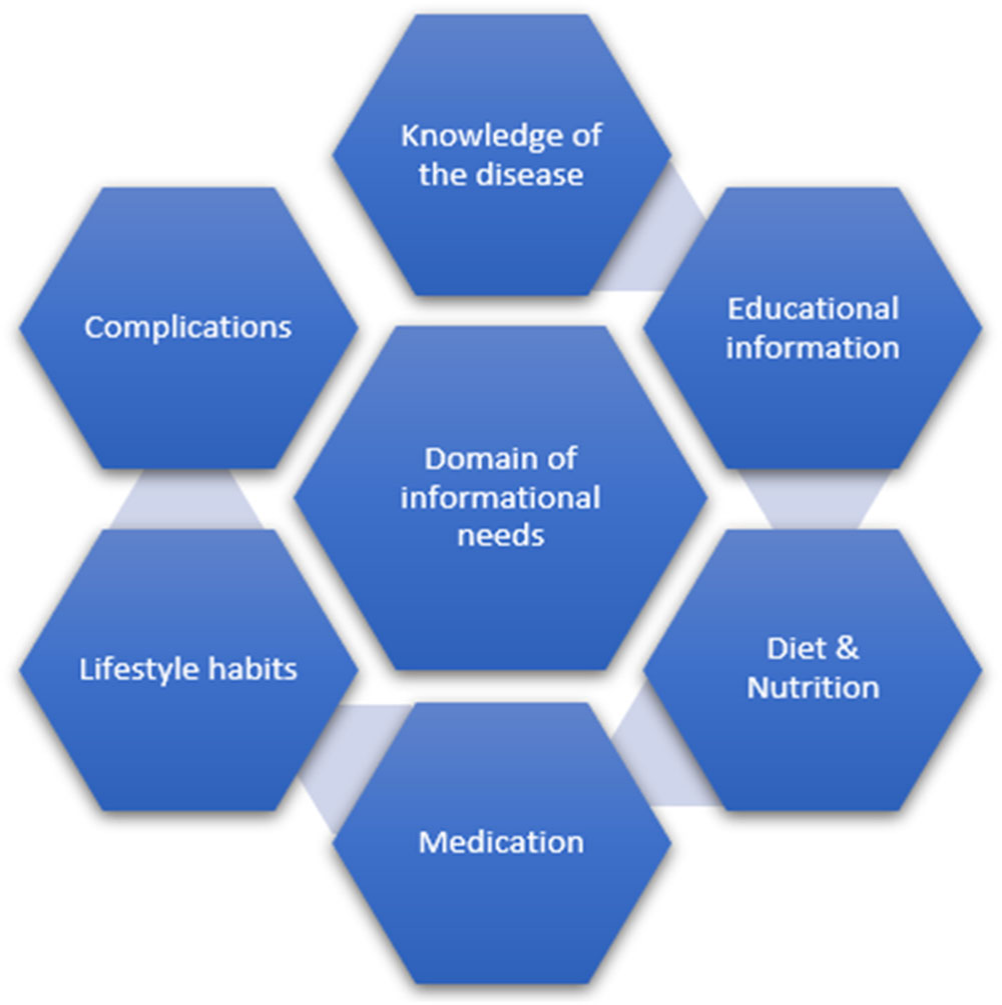
The Domain of informational needs of patients with IBD.

following the analysis of the patient's responses to the questionnaire, Knowledge of the disease, Medication, Educational information, Complications, Diet & Nutrition, and Lifestyle habits were the domain of informational needs of patients with IBD, respectively (Figure 1).

### 3.3 Essential Informational Needs of Patients With Inflammatory Bowel Disease

To investigate the differences in informational needs of patients with CD and UC, the questionnaire section of the essential informational needs of patients with IBD, including the Knowledge of the disease of patients with CD and UC (five questions), complications of the disease (three questions), Diet & Nutrition (four questions), Medications (three questions), Lifestyle habits (five questions), and educational information (five questions), were analyzed. After distributing the participants' answers, patients with CD and UC showed slight differences in informational needs (Table 2).

**Table 2:** Essential informational needs of a smartphone application for self‐management of patients with IBD.

Row	Domain	Essential informational needs	Crohn's disease (mean rating)	Ulcerative colitis (mean rating)	IBD (mean rating)
1	Knowledge of the disease (4.76)	About the disease	4.90	4.80	4.83
2		Common signs & symptoms	4.60	4.80	4.73
3		Therapeutic procedures	4.80	4.80	4.8
4		Prognosis	4.90	4.60	4.70
5		Diagnostic procedures	4.5	4.85	4.73
6	Educational information (4.50)	Stress management	4.55	4.55	4.55
7		Management of nutrition & diet	4.30	4.65	4.53
8		Self‐management strategies to prevent disease recurrence	4.65	4.75	4.72
9		Management of abdominal pain	4.25	4.27	4.26
10		Management of IBD‐related complications	4.35	4.47	4.43
11	Complications (4.56)	Risk of cancer	4.80	4.82	4.81
12		Complications & clinical manifestations	4.35	4.70	4.58
13		Routine tests to diagnose complications	4.25	4.30	4.28
14	Diet & nutrition (4.29)	Consumption of milk & proteins	4.25	4.22	4.23
15		Consumption carbohydrate	4.40	4.55	4.50
16		Consumption of fatty foods & fast food	4.10	3.95	4
17		Consumption of fruits & vegetables	4.65	4.32	4.43
18	Medication (4.41)	Adherence to medication	4	4.27	4.18
19		Type drugs	4.70	4.55	4.60
20		Side effects & medication interactions	4.25	4.55	4.45
21	Lifestyle habits (4.09)	Using the application	4.20	4.50	4.40
22		Having physical activity	3.45	3.70	3.62
23		Changing social habits	4.40	4.07	4.18
24		Appropriate exercise & physical activity	3.25	3.95	3.72
25		Use of mobile phones	4.45	4.57	4.53

*Note:* Each score shows the importance of the “Essential informational needs” in each item.

Responses were measured using a 5‐point Likert scale, ranging from “very important” to “unimportant.”

The scores of the items were as follows: 5 = very important, 4 = important, 3 = no idea, 2 = slightly important, and 1 = unimportant.

Patients with UC generally need more information regarding Knowledge of the disease, Complications, Medications, Educational information, and Lifestyle habits than patients with CD. Patients with CD generally need more information than patients with UC regarding Diet & Nutrition (Table 2). Patients with IBD were less interested in receiving information in the domain of Lifestyle habits and more interested in obtaining information in the domain of Knowledge of the disease.

### 3.4 The Required Capabilities of the Smartphone Application for the Self‐Management of Patients With IBD

A questionnaire was designed and reviewed in 15 items, including daily notes, reminders (medication, prescription renewal, doctor's appointment), Diet Menu, education (about the disease, treatment, medication side effects), psychological support (a report of the patient's feelings), Record Physical activity and exercises, Tracking appointments, test results, vitals, procedures, surgeries, and more, facilitating communication with healthcare providers, Transfer data or reports to healthcare providers, recording stimuli (such as food, stress, activity location, weather patterns, and genetics), audio and video files, video call, stool protocol, toilet finder, and password protection, to check the required capabilities of the self‐management application for patients with IBD (Table 3).

**Table 3:** Capabilities required the smartphone application for the self‐management of the patients with IBD.

No.	Items	Crohn's disease (*n* = 60), mean rating	Ulcerative colitis (*n* = 120), mean rating	IBD (*n* = 180), mean rating
1	Daily notes	3.50	3.20	3.30
2	Reminder (medication, prescription renewal, doctor's visit)	4	4.30	4.20
3	Diet Menu (food recommendations, meal plans, fluid intake monitoring, and recording meals)	4	4.27	4.18
4	Education (about the disease, treatment, and medication side effects)	4.65	4.65	4.65
5	Psychological support (a report of the patient's feelings)	3.95	4.60	4.38
6	Record Physical activity and exercises	3.40	4.20	3.93
7	Tracking appointments, test results, vitals, procedures, surgeries, and more	4.15	4.42	4.33
8	Facilitating communication with healthcare providers	4.30	4.62	4.51
9	Transfer data or reports to healthcare providers	4.50	4.47	4.48
10	Recording stimuli (such as food, stress, activity location, weather patterns, and genetics)	4.45	4.65	4.58
11	Audio and video files	3.90	4.32	4.18
12	Video call	3.30	3.67	3.55
13	Stool protocol	3.90	4.22	4.11
14	Toilet finder	3.90	4.37	4.21
15	Password protection	3.70	4.30	4.10

*Note:* Each score shows the importance of the “Essential informational needs” in each item.

Responses were measured using a 5‐point Likert scale, ranging from “very important” to “unimportant.”

The scores of the items were as follows: 5 = very important, 4 = important, 3 = no idea, 2 = slightly important, and 1 = unimportant.

According to the distribution of the participant's responses to the questionnaire, IBD patients were more interested in the education item (about the disease, treatment, and medication side effects), with an average score of (4.65). It was also determined, that patients were less interested in daily notes with an average score of (3.3), video calls with an average score of (3.55), and record physical activity and exercises with an average score of (3.93). Furthermore, patients with UC indicated a higher demand for the features necessary for self‐management applications than those with CD.

## 4 Discussion

In this research, an attempt was made to determine and identify the informational needs of patients (as end users) and collect all the essential items of informational needs in the design of a smartphone self‐management application. The findings of our research not only confirm and support previous studies but in addition to identifying these needs, the required capabilities of a smartphone application in the self‐management of inflammatory bowel disease were identified and determined.

Nowadays the importance of self‐managing IBD and its numerous complications—which significantly affect daily life—highlights the need for patient education in developing health apps for smartphones, specifically those aimed at increasing awareness and knowledge. Identifying the informational needs of patients with IBD and creating a framework to provide information in a way that fits the informational needs of these patients helps control better and manage this chronic disease. Improving healthcare services is undoubtedly only possible by using intelligent technologies and smartphone apps [25].

Karadag et al. highlights patients' dissatisfaction with the information provided and the importance of the informational needs of patients with IBD, affecting the understanding of healthcare providers and improving the quality of services [5].

The current study reveals that considering the age range of the patients interviewed, their educational background (with over 87% holding a diploma or higher), and their eagerness to engage with modern technologies such as smartphone health apps, IBD self‐management applications serve as a valuable resource for these individuals to enhance their understanding of their condition. our research suggests that patients with Crohn's disease (CD) and ulcerative colitis (UC) have different informational needs, which aligns with previous research [20].

The study also found that patients with UC patients need more information in the domains of Knowledge of the disease than patients with CD. Thus, patients with UC are more interested in obtaining information about their disease.

According to the present study, most of the participants with IBD are eager to have detailed information about their condition, such that the highest informational needs of patients with IBD are in the domain of Knowledge of the disease, suggesting that these patients lack sufficient knowledge about their condition.

Despite stating the objectives of the research and designing the questionnaire questions clearly and transparently, few participants chose the “no idea” option. It seems that these people were faced with uncertainty or lack of satisfaction about some questions, and for this reason, they chose the “no idea” option.

Designers and developers of self‐management applications should consider this issue and develop a product with appropriate educational content to meet the informational needs of patients with IBD [26].

The findings of our study showed that from the patient's point of view, items such as Education (about the disease, treatment, and medication side effects) (score 4.65 out of 5) and Recording stimuli (such as food, stress, activity location, weather patterns, and genetics) (score 4.58 out of 5) have a higher priority and importance than other items. Also, in the analysis of the responses of the participants, it was found that one of the capabilities required by these patients in using applications for self‐management of the IBD disease is the psychological support item (score 4.38 out of 5), which proves that these patients are more susceptible than others to mental illnesses and need psychological support.

Valerie Pittet et al.'s study [23] indicated that patients with CD have less anxiety over their daily lives than UC patients and are more satisfied with the information they receive on their disease. One of the items designed in the field of essential informational needs was about the importance of cancer risk information for people with IBD, most of the respondents expressed the desire to know more about their cancer risk, which confirms the concern of these patients about the spread of the cancer.

Given that patients with IBD are at an elevated risk for colorectal cancer, there is a heightened need to educate these individuals and to emphasize the critical importance of regular follow‐ups and cancer screenings.

Nowadays, patients with IBD can access doctors and other healthcare staff via the Internet and social media and find treatment centers for their disease. Nevertheless, the participants' responses in the field of required capabilities showed that communication between patients and their healthcare providers should be facilitated.

This study suggests self‐management apps designed for patients with IBD to facilitate this communication and meet their educational needs. The clinical results of this study indicate that individuals with a family history of IBD are at a greater risk of developing the condition, with over 87% of participants having a familial link to IBD. Consequently, educating at‐risk individuals and their families is crucial for preventing or mitigating disease‐related risks and complications.

Considering the importance of diet management in patients with inflammatory bowel disease, the need to develop nutrition monitoring and management strategies is felt. One of the most important tools for monitoring and managing nutrition and diet is Telenutrition.

Telenutrition, by using Telenutrition modules used on smart applications, can help patients with IBD in implementing the recommended diet [27].

Today, the use of new technologies and tools can help manage chronic diseases, including inflammatory bowel disease. Artificial intelligence is a valuable tool that can help patients with IBD in managing their condition [28].

The research by Majidova et al. [29] shows that techniques based on artificial intelligence can be the basis for personalizing the treatment of this disease and evaluating the response to a biological treatment. Also, by using complex calculations and approaches based on artificial intelligence, it is possible to help improve the diagnosis, treatment, monitoring, and prognosis of inflammatory bowel disease [29].

One of the emerging areas in the management of inflammatory bowel disease is the personalization approach that allows the customization of treatment based on treatment groups [30, 31]. Therefore, with the help of this field, by identifying the individual characteristics of the patient, the best treatment can be performed at the best time for the patient [32].

One of the strengths of the current research compared to similar studies is the presence of modules and personalized capabilities in the information needs and capabilities section of the self‐management mobile application for patients with inflammatory bowel disease. For example, in the diet menu, the presence of personalized features such as food recommendations, meal plans, fluid intake monitoring, and recording meals increases satisfaction and improves the quality of life of patients with inflammatory bowel disease.

In a study conducted by Yu et al. in South Korea [20] regarding the knowledge and informational needs of patients with IBD, patients had the most informational needs in the domain of Knowledge of the disease, confirming the present study's findings.

It seems that the use of smartphones, especially mobile self‐management applications, can play a role as an intervention tool in increasing participation and improving the quality of these patients and lead to an increase in communication between patients and health care providers.

So according to the review of the related literature and the needs assessment of the patients themselves, the present study investigated and identified the informational needs of patients with IBD in the design of self‐management applications for IBD.

## 5 Limitations

The study encountered some limitations. Initially, it was limited to a single hospital in Tehran, restricting the applicability of its conclusions beyond this setting. Furthermore, the research spanned four age categories, ranging from 18 to 60. Hence, its findings do not apply to individuals younger than 18. A third limitation is the comparatively lower occurrence and prevalence of CD compared to UC within Iran, resulting in a smaller sample size of participants with CD compared to those with UC.

## 6 Conclusion

In this study, the informational needs of patients with IBD were identified and determined to design the essential informational needs of the self‐management application for IBD. The present study's findings can be used to develop and evaluate self‐management applications for patients with IBD based on their informational needs. Accordingly, paying attention to the informational needs of these patients in developing self‐management applications for patients with IBD increases the level of knowledge, empowers the patients, improves the quality of life, and provides high‐quality healthcare.

## Author Contributions

**Farkhondeh Asadi:** supervision, conceptualization, methodology, writing–original draft, writing–review and editing. **Azamossadat Hosseini:** conceptualization, methodology, writing–original draft. **Amir Hossein Daeechini:** writing–review and editing, writing–original draft, data curation, formal analysis, investigation.

## Ethics Statement

This study was reviewed and approved by the review board and ethics committee of Shahid Beheshti University of Medical Sciences (IR. SBMU. RETECH. REC.1402.156). All methods were performed in accordance with the relevant guidelines and regulations. Informed consent was obtained from all participants involved in the study.

## Conflicts of Interest

The authors declare no conflicts of interest.

## Transparency Statement

The lead author Farkhondeh Asadi, Azamossadat Hosseini affirms that this manuscript is an honest, accurate, and transparent account of the study being reported; that no important aspects of the study have been omitted; and that any discrepancies from the study as planned (and, if relevant, registered) have been explained.

## Data Availability

The authors confirm that the data supporting the findings of this study are available within the article [and/or] its supplementary materials. All authors have read and approved the final version of the manuscript Farkhondeh Asadi had full access to all of the data in this study and takes complete responsibility for the integrity of the data and the accuracy of the data analysis.
